# Enhancing team development in an internal medicine resident continuity clinic

**DOI:** 10.1080/10872981.2024.2430570

**Published:** 2024-11-19

**Authors:** Majken T. Wingo, Andrew J. Halvorsen, Emily L. Leasure, Jocelyn A. Wallace, Jill M. Huber, Tammy R. Mathias, Kris G. Thomas

**Affiliations:** a Division of Community Internal Medicine, Geriatrics, and Palliative Care, Department of Medicine, Mayo Clinic, Rochester, MN, USA; b Internal Medicine Residency, Department of Medicine, Mayo Clinic, Rochester, MN, USA

**Keywords:** Interprofessional education, team development, TeamSTEPPS, continuity clinic, internal medicine resident education, safety attitudes

## Abstract

Interprofessional teamwork is important for the provision of safe, high value patient care and is recognized as essential by the ACGME. We aimed to assess the impact of an interprofessional continuity clinic teamwork curriculum on perceptions of team development and patient safety. This project was conducted in an IM Resident Continuity Clinic where 96 residents, supported by 28 faculty and 48 interprofessional team members, attended continuity clinic two afternoons per week during alternating months of a 50/50 outpatient-inpatient training model. Teams were configured into two groups of residents, faculty and interprofessional team members. The randomly selected intervention group participated in strategically-timed TeamSTEPPS training. The control group received usual clinic education. Teamwork and safety climate were measured using the Team Development Measure (TDM) and Safety Attitudes Questionnaire (SAQ) collected before and after the intervention. Following the teamwork curriculum, team development improved in the intervention group as compared to control [mean change (95% CI) +13.9 (+9.3, +18.6) versus + 4.8 (+0.4, +9.1), *p* = 0.007]. Though 30% of the individual items on the SAQ improved significantly in the faculty intervention group as compared to control, the overall improvement in SAQ [intervention mean change + 0.4 (+0.2, +0.5), control mean change + 0.2 (−0.1, +0.5)] was not statistically significant (*p* = 0.36). It is feasible to implement a TeamSTEPPs-based interprofessional teamwork curriculum among IM residents in a block clinic model and achieve enhanced teamwork and safety attitudes. Additional assessment of clinical and educational outcomes is ongoing.

## Introduction

The provision of high quality medical care is a steadfast goal of clinical practice, but the best method to teach trainees how to provide such care remains a matter of debate [1]. National initiatives have emphasized the importance of interprofessional education (IPE) to enhance collaboration and communication in healthcare teams [2]. The Accreditation Council for Graduate Medical Education (ACGME) has made patient safety and health care quality two of its six focus areas for the Clinical Learning Environment Review (CLER), which is required for training program accreditation [3]. In addition, the American Boards of Family Medicine, Internal Medicine (IM), and Pediatrics have promoted interprofessional education to enhance team performance through Professionals Accelerating Clinical and Educational Redesign (PACER), a professional development program.

While accreditation bodies recognize the importance of IPE to improve teamwork, much of the research among trainees has been performed using simulation or workshops [4–10]. There is a paucity of research conducted among medical trainees utilizing IPE to enhance teamwork, quality, and safety in the clinic itself. Furthermore, projects undertaken with trainees in block scheduling models have been underrepresented in the IPE literature, particularly in the outpatient setting, due to difficult scheduling of trainees in the clinic. Teamwork competencies to improve team function in the education clinic have been proposed, but the optimal mechanism by which to teach those competencies remains undefined [11]. The Agency for Healthcare Research and Quality (AHRQ) has designed and tested an office-based Team Strategies and Tools to Enhance Performance and Patient Safety (TeamSTEPPS), which has been effective to improve team communication, reduce error, and improve patient satisfaction [12]. In the education clinic setting, TeamSTEPPS implementation has been associated with improved patient satisfaction, patient safety perceptions, and resident perceptions of teamwork [13]. Despite prior literature in these domains, there is insufficient information regarding how implementation of ambulatory TeamSTEPPS may impact team development and patient safety in an education clinic with a block scheduling model and discontinuous resident presence.

Due to duty hour changes and resident preferences, scheduling models that prioritize blocks of inpatient or outpatient clinical duties have been increasingly adopted by IM residency programs [14]. Various block models have been described and are often referred to as X + Y models where X reflects the number of weeks residents rotate on inpatient services and Y represents the number of weeks of ambulatory service including continuity clinic [15]. Though residents are generally more satisfied with this schedule, consequences of X+Y models have shown variable impacts on continuity, patient satisfaction, and teamwork [14,16]. X + Y scheduling models present a significant challenge when interprofessional education endeavors are pursued in continuity clinic because of discontinuous resident presence. For residency programs with X + Y blocks of 4 weeks +4 weeks (also referred to as 50:50 block models), effective team development and IPE may be particularly challenging due to prolonged time away from clinic while on inpatient services.

The optimal way to enhance team development and improve patient safety in a resident continuity clinic is not known. We sought to improve teamwork using an existing TeamSTEPPS framework with proven efficacy to improve team performance [17]. We aimed to improve team development by IPE designed to learn TeamSTEPPS competencies together as a multidisciplinary team. In doing so, we hypothesized that improved team development would enhance patient safety and improve metrics of patient care quality.

## Methods

This project was conducted in an IM Resident Continuity Clinic at Mayo Clinic in Rochester, Minnesota during the 2017–18 academic year where 96 residents, supported by 28 faculty and 48 interprofessional team members, attended continuity clinic two afternoons per week during alternating months of a 50:50 outpatient-inpatient training model [18]. In this 50:50 model, residents had continuous blocks of 4 week inpatient rotations alternating with 4 week outpatient rotations during which they had continuity clinic two afternoons per week. This continuity clinic site was chosen as part of the PACER national initiative, which fostered professional development of team leaders for educational clinic changes aiming to improve interprofessional education and teamwork [19]. Clinic teams were configured into two groups of residents, faculty, and interprofessional team members assigned to work together in distinctly co-located clinical areas. Teams were block randomized into intervention and control groups.

At baseline, there was no teamwork curriculum within the resident continuity clinic. The residents’ baseline communication curriculum focused on patient communication without specific instruction on interprofessional communication. Change leaders for the intervention group attended a TeamSTEPPS for Office-Based Care 2.0 Master Trainer Course prior to implementation [20]. The intervention group participated in iterative in-clinic IPE sessions involving the multidisciplinary clinical team. Participants included IM residents in all years of training, continuity clinic faculty physicians, licensed practical nurses (LPNs), registered nurses (RNs), clinical pharmacists, medical administrative assistants, clinical assistants, patient appointment schedulers, and social workers. Average group size was 20 participants per session, which were broken into 3–5 small groups depending on the activity. The first two sessions focused on team building activities that emphasized learning the individuals on the team, understanding the roles of team members, and flattening hierarchies within the team. Subsequent sessions each focused on a TeamSTEPPS theme such as closed loop communication, Situation-Background-Assessment-Recommendation/Request (SBAR) communication, structured handoffs, team brief principles, mid-clinic huddle principles, debrief principles, and safety language (such as the Concerned-Uncomfortable-Safety issue [CUS] framework). Sessions started with a group PowerPoint describing the theme, and then small groups explored the theme with clinical cases, root cause analyses of sentinel events, and group quizzes with the goal of fostering team development and improving perceptions of patient safety.

The intervention group participated in ambulatory TeamSTEPPS training in a unique scheduling format that allowed for weekly attendance during the first 60 mins of clinic during one of residents’ two continuity clinic afternoons per outpatient block. During outpatient blocks, residents had continuity clinic two afternoons per week in one of the following schedules: Monday&Wednesday, Tuesday&Thursday, Wednesday&Friday, Monday&Tuesday, Monday&Thursday, or Tuesday&Friday. To capture as many residents as possible during outpatient blocks, IPE sessions occurred on Monday, Tuesday, and Wednesday of the first two blocks of residency training (allowing participation of residents that started the academic year on an inpatient block). If a resident was available to participate in TeamSTEPPS training more than once per week, they were asked to attend only one of the sessions.

After participation in ambulatory TeamSTEPPS, the intervention group implemented daily pre-clinic briefs with the multidisciplinary team to discuss staffing, appointment access, anticipated patient needs, and anticipated delays for the clinical afternoon. Later in the academic year, the intervention group participated in an interprofessional workshop focused on team engagement where the concepts of Plan-Do-Study-Act (PDSA) cycles were discussed. The control group received usual continuity clinic education; supervisory physicians teaching residents at the point-of-care when evaluating and managing patients seen in resident continuity clinic. Control group patient care was supported by and equivalent number and variety of care team members as the intervention group, but there was no interprofessional instructional content.

Team development was measured using the Team Development Measure (TDM), a 31-item instrument with validity evidence measuring team cohesion, communication, roles/goals clarity, and team primacy [21]. Safety climate was measured with a 13-item version of the Safety Attitudes Questionnaire (SAQ) [22]. Surveys were given before and after the intervention. Team processes were measured by items that indicate engagement of clinical teams: room light utilization (residents turning on ‘provider in’ indicator lights when starting a visit), resident ordering of a nurse-driven protocol for management of uncontrolled hypertension, and patient wait time. Room light utilization was measured by the exam room light system, and information was collected by randomly sampling 10 visits from each week of the intervention. Residents who turned on the ‘provider in’ light at the beginning of the visit were counted as adhering to room light utilization. Ordering of hypertension protocol was tracked manually in control and intervention groups. Automated reports within the electronic medical record tracked patient check-in times, and patient check-in time to ‘provider in’ light illumination calculated wait time. Patient wait times were calculated from the later of report time (20 minutes prior to scheduled appointment) or actual check in time to avoid penalizing teams for early check ins. Visits that did not use the ‘provider in’ light were excluded. Educational outcomes were measured by resident-of-rotation and faculty-of-rotation evaluations. The interprofessional education sessions were not evaluated separately. Patient satisfaction was measured by the American Board of Internal Medicine Patient Assessment Module questions [23,24]. Patient outcome metrics such as vaccination rates, preventive screening, blood pressure control, and hemoglobin A1c were collected before and after the intervention.

Data analysis used the Cochrane-Armitage trend test for TDM and SAQ. Measures of clinical team process utilization were summarized using descriptive statistics, and statistical significance for light indicator use and patient wait time was assessed using generalized linear models estimated via generalized estimating equations with an exchangeable covariance structure to account for repeated appointments by individual physicians. Educational and patient outcomes were analyzed using Fisher’s Exact Tests, t tests, or ANOVA, as appropriate. A statistical significance threshold of 0.05 was used for all comparisons, with analyses conducted using SAS statistical software (Version 9.4, SAS Institute Inc., Cary, NC, USA).

The Mayo Clinic Institutional Review Board reviewed the protocol and deemed this project exempt from review.

## Results

All participants were invited to complete both the TDM and SAQ. Survey response rates on TDM and SAQ were 122/172 (71%) pre-intervention and 126/172 (73%) post intervention. Team development improved in the intervention group as compared to control [mean change (95% CI) +13.9 (+9.3, +18.6) versus + 4.8 (+0.4, +9.1), *p* = 0.007]. The overall improvement in SAQ [intervention mean change + 0.4 (+0.2, +0.5), control mean change + 0.2 (−0.1, +0.5)],) was not statistically significant (*p* = 0.36), but 30% of the individual items on the SAQ improved significantly in the faculty intervention group as compared to control. Items with significant improvement in the faculty intervention group included: 1) The culture in the clinical area made it easy to learn from the errors of others [mean (SD) +0.67 (0.71), *p* = 0.02]; 2) The physicians and nurses here work together as a well-coordinated team [mean (SD) +0.67 (0.87), *p* = 0.05]; 3) It is easy for personnel to ask questions when there is something that they do not understand [mean (SD) +0.56 (0.53), *p* = 0.01]. Faculty also felt that teamwork overall was better when compared to the control group [mean change (SD) +0.56 (0.71), *p* = 0.05].

Clinical team process utilization measures showed improvement in various domains. For the six months of 10 appointment weekly samples per side, the intervention group used the ‘provider in’ light for 243/252 (96.4%) appointments, while the control used it for 222/254 (87.4%) appointments. The odds of using the light were 4.2 times higher in the intervention group compared to the control (99% CI 1.5 to 11.5; *p* = 0.005). Also from the 6-month sampling data, the mean wait time was estimated to be 10.9 minutes shorter in the intervention group compared to control (99% CI 7.0 to 14.8; *p* < 0.01). During the academic year, 13 residents in the intervention group ordered a nurse-driven protocol for management of uncontrolled hypertension compared to 3 residents in the control group. There were no statistically significant differences in rotation evaluations, patient satisfaction, or patient outcomes. There were no statistically significant differences when analyses were performed examining resident characteristics such as gender or post-graduate training year.

## Discussion

In this evaluation of internal medicine resident continuity clinic team development, we found that teamwork and clinic process efficiency can be improved by utilizing interprofessional education supported by a clinic-based TeamSTEPPS framework. Patient safety climate may also be enhanced by this training. While our findings confirmed some previous research of TeamSTEPPS training in the academic primary care setting [13], to our knowledge, this is the first intervention conducted among IM residents in a block scheduling model. Other IM residency programs may be struggling with the feasibility of providing interprofessional education amongst IM residents who have a discontinuous presence in continuity clinic. By providing a staggered scheduling approach, we ensured that all residents would have the opportunity to participate in at least one session per week. By holding these interprofessional education sessions for two consecutive 4-week blocks, we ensured that all IM residents were able to participate even if their first rotation of the year was in the inpatient setting. We have provided a framework here that has been successful in enhancing team development in this model, and we hope this finding is useful for other programs with similar block schedules.

Some safety attitudes improved significantly more amongst the faculty intervention group. Faculty felt that the culture in the clinical area made it easy to learn from the errors of others, that physicians and nurses worked together as a well-coordinated team, and that it was easy for personnel to ask questions. It is unclear why faculty physicians in the intervention group had greater improvement in safety attitudes than the rest of the clinical team. Prior studies have shown that faculty physicians may be more likely to acknowledge medical error [25]. It is also possible that faculty doctors may be more likely to understand key concepts of patient safety and thus serve as leaders in this domain [26]. Faculty engagement in patient safety attitudes may be an essential step toward incorporation of this important domain in IM resident continuity clinic education, and further study into the relationship between faculty and resident patient safety attitudes may elucidate the cause of these findings.

We saw a significant increase in team process utilization amongst the intervention group. Residents in the intervention group were more likely to order a nurse-driven protocol for managing uncontrolled hypertension. This protocol requires an electronic order and a warm handoff between the physician and the RN. It is likely that residents who learned about such team processes alongside nurses and then discussed those processes at preclinic team briefs were more frequently reminded about the availability of such processes and thus more likely to use them. While the overall measures of blood pressure control did not change in the intervention group, previous studies of nurse-driven protocols for hypertension management showed promise to improving patient outcomes with consistent use [27,28]. Our intervention was not specifically targeted to affect blood pressure outcomes, but future interventions could be considered. Efforts to enhance team-based care through the use of team processes for chronic disease management are ongoing.

Residents who participated in TeamSTEPPS alongside nurse colleagues were more likely to use a ‘provider in’ room usage indicator light. Verbal reminders at the start of every half day during a preclinic team brief likely played a role. Improved room indicator light use may have contributed to team efficiency by allowing for situation awareness of patient/provider location and more rapid turnover of exam rooms between appointments [29]. In addition, better room light utilization may have contributed to the lower patient wait time seen in the intervention group since improving exam room availability has been shown to improve this metric [30]. Further research could explore whether improved team communication, improved situation awareness, or improved team efficiency contributed to these changes.

The outcomes of TeamSTEPPS interprofessional education amongst these teams have several limitations in interpretation. While resident and faculty physicians consistently practiced within their assigned groups, that was not the case for some nonphysician team members. For example, there were times when one of the groups was understaffed, and a team member (e.g., a nurse) from the intervention group may have practiced on a given afternoon with the control group team (and vice versa). This may have resulted in some contamination of results ascertained between the invention and control arms, which could limit the ability to detect differences between groups. In addition, to accommodate interprofessional education time, the intervention group residents saw one fewer patient per afternoon on days when they participated in IPE. This could have theoretically impacted patient-specific education, but since no difference was seen in patient outcomes, we do not believe that patient-specific learning was significantly altered by the intervention. Our timeframe for intervention and assessment was intentionally discontinued at the end of the academic year due to implementation of a new electronic medical record that significantly changed workflows and team dynamics. The impact of limited duration of intervention and assessment remains unclear. It is plausible that longer duration of intervention and assessment may be needed to enhance learning, demonstrate improvement, and to detect durable outcomes.

Interprofessional education of a clinic-based TeamSTEPPS framework is feasible within programs that have a 50/50 block scheduling model and may lead to improved team development and patient safety attitudes. Acknowledging the limitations of this study, the intervention provides one approach for fostering teamwork in an internal medicine resident clinic and may stimulate ideas for development of similar interventions in other institutions with discontinuous resident presence. We hope that this framework furthers the recommendations from CLER and ACGME and allows other internal medicine residencies to embrace IPE around ambulatory team-based care. Future studies and further team development education should focus on improving patient outcomes through these interventions.

## Data Availability

The data that support the findings of this study are available from the corresponding author [MTW] upon request.
